# Effect of Corrosion on the Bond Behavior of Steel-Reinforced, Alkali-Activated Slag Concrete

**DOI:** 10.3390/ma16062262

**Published:** 2023-03-11

**Authors:** Yifei Cui, Shihao Qu, Kaikai Gao, Biruk Hailu Tekle, Jiuwen Bao, Peng Zhang

**Affiliations:** 1 Center of Durability & Sustainability Studies of Shandong Province, Qingdao University of Technology, Qingdao 266000, China; 2 The Second Engineering Company of China Railway No. 14 Engineering Bureau Group, Taian 271000, China; 3 Institute of Innovation, Science and Sustainability (IISS), Federation University Australia, University Drive, Mt Helen, Ballarat, VIC 3350, Australia

**Keywords:** alkali-activated concrete, steel bar, pull-out test, steel corrosion, bond strength

## Abstract

Alkali-activated slag concrete (ASC) is regarded as one of the most promising sustainable construction materials for replacing ordinary Portland cement concrete (OPC) due to its comparable strength and outstanding durability in challenging environments. In this study, the corrosion of steel bars embedded in ASC and OPC was studied by means of an electrically accelerated corrosion test of steel bars in concrete. Meanwhile, the bond performance of the corroded steel bars embedded in ASC was tested and compared with corresponding OPC groups. The results showed that ASC and OPC behaved differently in terms of bond deterioration. The high chemical resistance of ASC decreased the corrosion of steel bars and, thus, increased the residue bond strength and the bond stiffness.

## 1. Introduction

The scale of the construction industry has made it one of the leading contributors to greenhouse gases. The greenhouse gases released and the fossil fuels consumed in the production process of Portland cement have been regarded as the main sources of pollution. The per capita cement production is higher than the per capita food consumption per year, and increasing future demand is expected [1]. With its 4.1 billion metric tons of cement production, the cement industry alone accounts for the highest proportion of global anthropogenic CO_2_ emissions [2]. Thus, research into and application of sustainable cementitious materials is one of the most important goals in building materials to promote green development.

To comply with the trend of sustainable development, the exploration and development of cementitious materials with low pollution and energy consumption are extremely important. Alkali-activated or geopolymer materials, as the focus of sustainable cementitious materials, have attracted the attention of scholars worldwide due to their advantages in energy savings, environmental friendliness, and outstanding durability [3,4]. Studies have shown that avoiding the high direct emission of CO_2_ from cement production and reducing the process energy can make this binder’s emission 5–6 times lower than cement [5]. In addition to environmental benefits, ASC alkali-activated materials also offer good chemical resistance, bond behavior, frost resistance, and thermal shock and/or fire resistance [6,7].

The outstanding engineering properties of alkali-activated concrete (ASC) give them excellent application potential. However, it is difficult to get a conclusive remark on their different properties due to the various types of alkali-activated materials, depending on their source materials, activators, and curing methods. One such variation of alkali-activated material is ASC. This is produced by activating ground-granulated blast-furnace slag (GGBS) with alkaline solutions, such as sodium silicate and sodium hydroxide. ASC has good mechanical behaviors [8] and durability [9]. Meanwhile, there is a limited understanding of the corrosion resistance of reinforced ASC due to a lack of data.

The corrosion of reinforcement is one of the most important durability problems for reinforced concrete structures [10,11]. The corrosion of steel reinforcements under marine environments has costed the world billions annually [12]. Studies on the corrosion behavior of steel bars embedded in ASC under a chloride ingress environment showed promising results. Chaparro et al. [13] investigated the corrosion development of steel-reinforced ASC and OPC concrete. Their results showed that after three months of immersion in 3.5% NaCl solution, the steel bars showed similar corrosion behavior when embedded in ASC and OPC concrete. Holloway and Sykes studied the corrosion of mild steel in ASC mortars containing NaCl admixtures [11]. Their results showed that despite the aggressive environments and very high concentrations of chloride admixture, only a low level of corrosion was encountered. Criado and Provis found that rebars embedded in alkali-activated mortar immersed in an alkaline chloride-rich solution showed no evidence of corrosion [14]. Shi et al. [15] reported better protection of embedded steel in ASC compared to OPC after chloride exposure. Nabeel experimentally illustrated the mild bond deterioration between corroded steel bar and ASC [16].

While many studies reported the superior corrosion resistance of ASC over OPC, others indicated an opposite view [17]. This calls for further investigation of the corrosion behavior of steel-reinforced ASC. Furthermore, there is limited or no research on the effect of corrosion on the bond performance of steel-reinforced ASC. Thus, there is a strong demand for more thorough scientific and analytical work to determine the bond mechanisms in order to better understand and control the damage caused by steel corrosion in ASC.

In this study, an electrically accelerated corrosion test was used to create and compare the corrosion condition of steel bars embedded in ASC and OPC. The bond deterioration caused by the corrosion between the steel bars and concrete was also studied using pull-out test specimen.

## 2. Experiments

This research investigated the corrosion behavior and bond performance between steel bars and ASC and OPC concretes.

### 2.1. Materials and Mix Proportions

OPC and ASC concretes were used. A hot-rolled, low-carbon ribbed steel bar with a yield strength of not less than 500 MPa (HRB500) and a nominal diameter of 16 mm was used [18]. Grade PO42.5 cement and GGBS were used for the OPC and ASC concretes, respectively. The chemical composition of the cement and the GGBS are shown in Table 1. A fine aggregate with a particle size of 0.125–5 mm and a coarse aggregate with a 5–30 mm particle size range were used for both OPC and ASC concretes. The activator solution used for the ASC concrete was a mixture of sodium silicate and sodium hydroxide. The sodium silicate was obtained from Linyi Green Chemical Co., Ltd. (Linyi, China), and the sodium hydroxide of laboratory purity was obtained from Shanghai Epiphanius Chemical Reagent Co., Ltd. (Shanghai, China) The sodium hydroxide was a white irregular flake. The properties of the sodium silicate and sodium hydroxide are shown in Table 2 and Table 3.

The mix proportion for the OPC and ASC concretes is shown in Table 4. The water glass used had a modulus of 1.8 (the molar mass of Na_2_O/SiO_2_) and an alkali equivalent (the mass fraction of Na_2_O/ slag) of 7%. The alkali solution was prepared 24 h in advance and left in a controlled-environment room (ER, 20 ± 1 °C, 98% humidity).

Specimens for compressive strength and accelerated corrosion tests were prepared and cured in the controlled-environment room for 28 days. The compressive strength of the concretes was tested according to the standard [19], and the test results are shown in Table 4.

### 2.2. Electrically Accelerated Corrosion Test of Steel Bars

#### 2.2.1. Selection of Corrosion Current Density

Under natural conditions, the current density of corrosion on the surface of steel bars in concrete ranges from 0.1 to 10 μA/cm^2^ [16]. To be close to the corrosion condition under natural conditions, a similar density was selected for the accelerated steel corrosion apparatus, and the general current density range was from 100 to 1000 μA/cm. In the pre-test of the accelerated corrosion of the steel bar, it was found that the current density of the steel bar in alkali-activated slag concrete could not meet a stable current density, and the designed corrosion current density could not be obtained even if the voltage at both ends of the steel bar was increased. Therefore, the equivalent initial current density was selected for the test; a 0.2 mA/cm^2^ density was selected as the initial current density, the corrosion degree of the steel bar was calculated by recording the change in current intensity in real time, and the corrosion degree of the test piece was 0.5%, 1%, 2%, and 3%.

#### 2.2.2. Test Procedure for Electrically Accelerated Corrosion of Steel Bars

**Figure 1:**
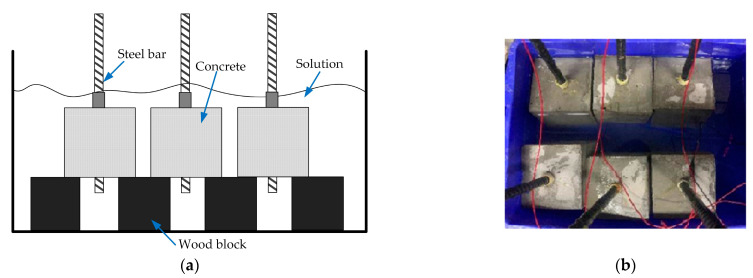
Specimen preparation: (**a**) soaking diagram and (**b**) connection details.

**Figure 2:**
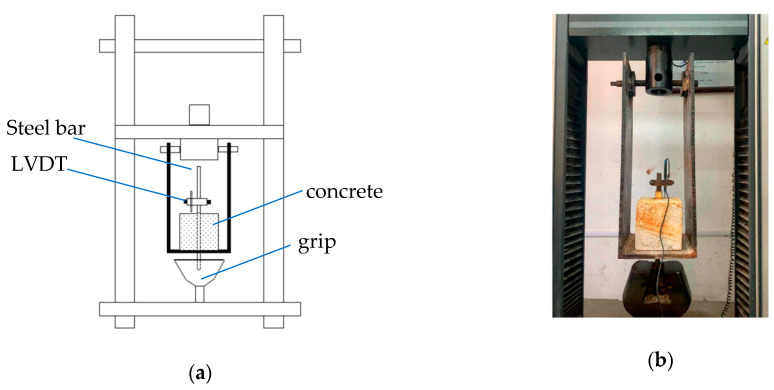
Bond test: (**a**) schematic test diagram and (**b**) actual setup.

A 5% sodium chloride (NaCl) solution was selected as the electrolyte solution. To reduce the resistivity of concrete and obtain a better corrosion effect after 28 days of curing with the test piece, the test piece was immersed in the electrolyte solution for 7 days, and the height was adjusted with a test block at the bottom of the test piece, as shown in Figure 1. After the test piece was soaked for 7 days, the wires were connected. Since the wire connection mode did not affect the test results, a parallel connection mode was adopted for convenience. A schematic diagram of the wire connection is shown in Figure 2. The positive pole of the power supply was connected to the steel bar, and the negative pole was connected to the inert electrode. The power was connected to ensure the alkali-activated concrete specimen and the ordinary concrete specimen had the same initial current intensity. The current intensity of the concrete samples was recorded instantly. Additional power was applied to the concrete samples with the same voltage at both ends, and the change in voltage intensity was recorded.

The mass change in the reinforcing steel bar due to corrosion was calculated by applying Faraday’s Law, as shown in Equation (1) [20].

(1) $$\Delta m=\frac{MIt}{ZF}\;,$$

where ∆*m* is the mass loss in the steel bar; *M* is the molar mass of iron (56 g/mol); *I* is the electric current (A); t is time (sec); *Z* is the valency number of ion of iron taken as 2; and *F* is the Faraday constant (96,485.3 Coulombs/mol).

### 2.3. Pull-Out Test and Measurement of Actual Corrosion

After the specimens reached the designed corrosion degree, a central pull-out test was carried out. The test equipment includes a universal testing machine, a homemade loading rack, an LVDT displacement meter, a data acquisition box, and a notebook computer. This universal testing machine is mainly used to apply a pull-out force at the loading end of a steel bar. The homemade loading frame is welded by three steel plates with a thickness of 1.5 cm. It is mainly used to fix the test piece and ensure that the test piece is in the center. The LVDT displacement meter and the data acquisition box are used to measure the slip distance at the free end of the steel bar, and the laptop is mainly used to export the test data. In addition, the loading device and loading method used in this test have been authorized for the utility model patents of China.

The central pull-out test was carried out according to the provisions of the standard for test methods of concrete structures, and the loading speed was 2 mm/min [21] A schematic diagram of the pull-out test and the real experimental setup are shown in Figure 2.

After the pull-out test of the corroded specimens, the steel bars were removed, and the concrete in the bonding area was cut off with an angle grinder. The steel bars were then brushed, cleaned, and then weighed. After calculating the clean mass (*m*_0_) and the corroded mass (*m_c_*) of the steel bars, the actual corrosion degree of the steel bars was calculated using Equation (2) [22]:

(2) $$\Delta m_{r}=\frac{m_{c}-m_{0}\;\;}{m_{0}},$$

where ∆*m_r_* is the corrosion degree.

## 3. Results and Discussion

### 3.1. Corrosion Degree of Steel Bars

After the pull-out test, the reinforced bars were taken off and the bars inside ASC and OPC showed different degrees of corrosion.

**Figure 3:**
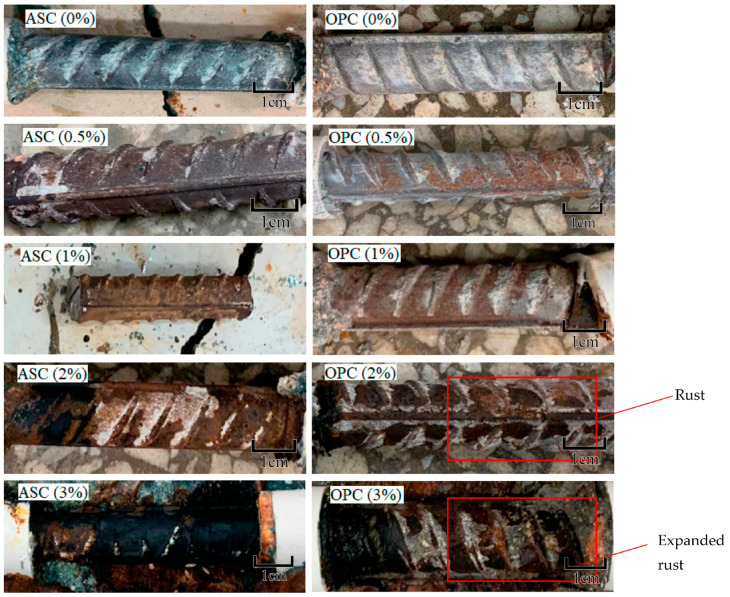
Steel bar corrosion in ASC and OPC at different designed corrosion degrees.

**Figure 4:**
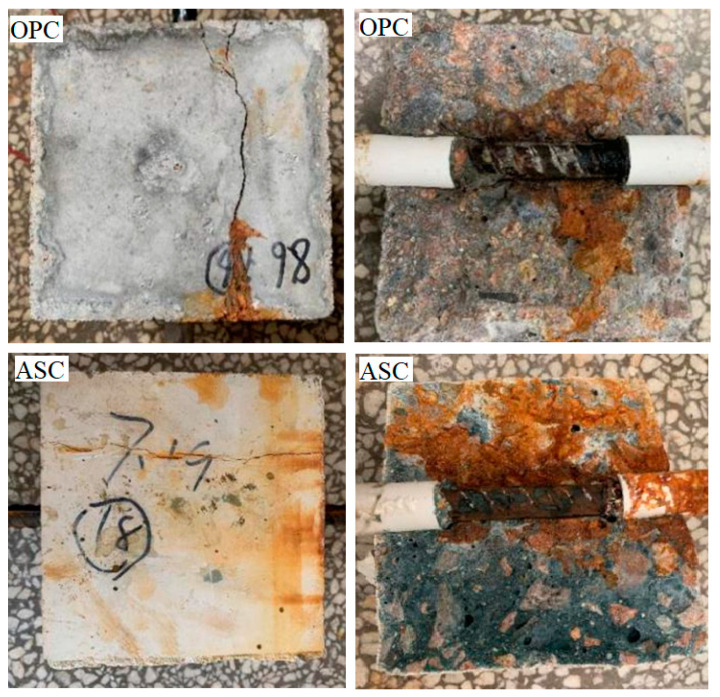
OPC and ASC corrosion cracking.

With the extension of corrosion time, the surface of the steel bars gradually develops from pitting corrosion to the whole surface of the steel bars, as shown in Figure 3. It can be seen in the figure that the corrosion products of the steel bars gradually increase with increasing corrosion degree. During the accelerated corrosion test, ASC and OPC showed the phenomenon of rust expansion and cracking at different designed corrosion degrees. The corrosion morphology and cracking are shown in Figure 4.

**Figure 5:**
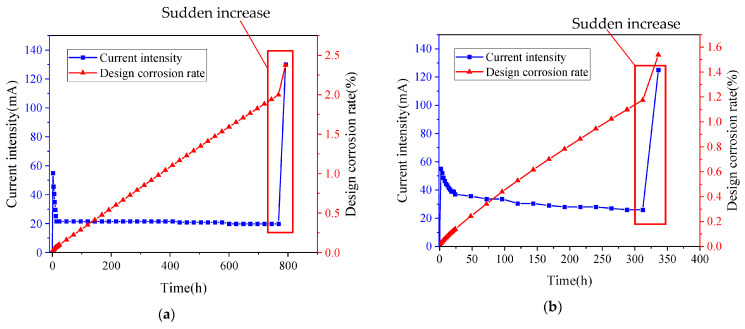
Relationship between the current intensity and designed corrosion degree: (**a**) ASC and (**b**) OPC.

Figure 4 shows the consequence when the expansion produced by the corrosion products have exceeded the tensile strength of the concrete. The expansion stress exceeded the tensile strength of the concrete, resulting in cracks that distributed along the corroded bar. During the test, the relationship between the designed corrosion degree and the current intensity with time was obtained, as shown in Figure 5. From the ordinate in the figure, it can be seen that the current intensity of both ASC and OPC specimens gradually decreases with the progress of the test and finally tends to become flat. The corrosion degree of the steel bars also gradually increases. The sudden increase in current intensity is due to the cracking of the test block caused by the corrosion of the steel bars, which reduces the resistance of the concrete and increases the current intensity dramatically. The abscissa in the figure shows that the differences in cracking time between the ASC and OPC specimens. This is caused by the different current densities in different concretes, but the corrosion state of the two kinds of concrete can be judged from the designed corrosion degree. For the ASC specimen, corrosion cracking occurs at a designed corrosion degree of 2%, while for the OPC, it occurs at only 1.2%, showing that ASC provides stronger hoop stress to the steel bar and delays the cracking of the specimen [16].

Table 5 shows the designed value and the actual measured value of the corrosion degree of the steel bars embedded in ASC and OPC. The * in the table represents the cracking of the test piece due to corrosion. It can be seen that the designed value and the measured value of the corrosion degree of the steel bars embedded in ASC and OPC show some differences. The reason for the difference is that in the case of the designed corrosion degree, it is calculated based on Faraday’s law, which assumes general corrosion, while in the case of the actual corrosion degree, there is some localized corrosion. Nguyen and Lambert also observed a similar difference between actual and theoretical mass losses [22]. Another phenomenon that needs to be explained is that at higher corrosion degrees, the designed rate is less than the measured value. This is because the corrosion current can directly act on the surface of the steel bars through the crack to accelerate the corrosion of the steel bars.

Apparently, the average corrosion degree of the steel bar embedded in ASC is lower than that the steel bar embedded in OPC. During the whole accelerated corrosion period, the current density of ASC is constantly lower than that of OPC. The superior corrosion resistance of ASC is due to its considerably dense microstructure. The content of permeable pores in ASC is much lower than that in OPC, which provides ASC a great anti-permeability [23,24]. Additionally, the products of alkali-activated slag (C-A-S-H) have been reported to have a better ability in terms of chloride solidification [25,26], which will reduce the concentration of free chloride ions inside the pore of concrete, as well as the degree of corrosion.

Meanwhile, it can be observed that for the ASC specimen, corrosion cracking occurs when the designed corrosion degree is 3%, while this rate for the OPC specimen is 2%. The good anti-crack ability provides ASC a better bond with the steel bar [27,28] and a superior durability [5].

### 3.2. Failure Modes of the Bond Test

**Figure 6:**
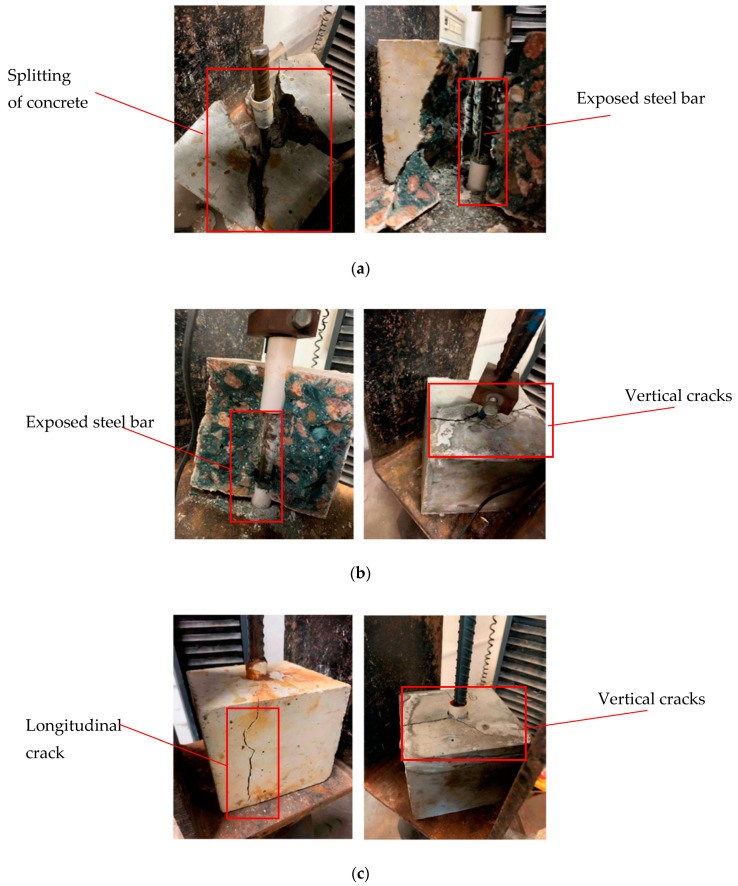
Failure modes of the test piece: (**a**) concrete collapse; (**b**) concrete splitting; and (**c**) pull-out splitting.

**Figure 7:**
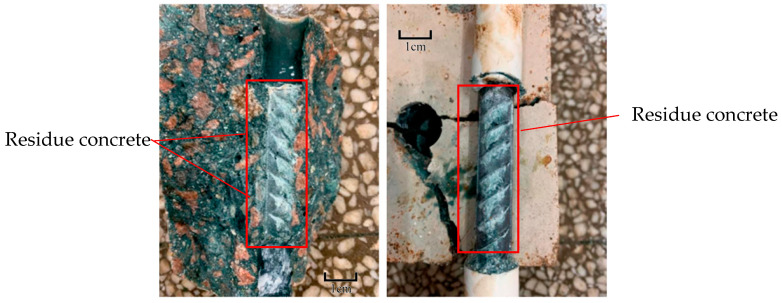
Concrete residue on the bar ribs.

From the pull-out test, it was observed that brittle splitting failure occurred when the test piece was not corroded and cracked. In the case of the specimens with corroded steel bars and cracked concrete, the steel bar is pulled out along the splitting crack. Figure 6 shows the failure modes of the specimens. With the progress of loading, the pull-out force increases. After the maximum pull-out force, the free end of the steel bar slides for a short distance, which is accompanied by cracking. After the failure, an observation of the concrete–steel interface reveals typical damage. Concrete pieces are found between the transverse ribs of the steel bar. As shown in Figure 7, the residue concrete in front of the ribs indicates the strong adhesion between ASC and steel.

Although the failure mode of the specimen in this test is a brittle splitting failure, the failure phenomenon is different with increasing corrosion degree. At lower corrosion degrees, there is no obvious crack on the surface of the test piece as the failure is approached. The specimen then splits without any warning, accompanied by a violent sound. Figure 6a shows the specimens from such failures. It is believed that the corrosion products on the surface of the steel bar fill the pores of the bonding interface between the concrete and the steel and enhance the bonding strength [16]. When the steel bar is pulled, it must overcome the stronger circumferential stress. With an increase in the corrosion degree of the steel bar, the phenomenon of splitting failure of the specimen also changes. When the pull-out load reaches the maximum value, the surface of the specimen cracks, and splitting failure occurs after the specimen sends out a rapid bursting sound. However, there is no collapse, as shown in Figure 6b. This is because, after the corrosion degree increases, the corrosion products increase, and the volume increases, destroying the contact interface between the two and reducing the concrete’s circumferential stress on the steel bar. The test piece does not undergo severe damage when the steel bar is pulled out. When the expansion force generated by the corrosion products causes the surface of the test piece to exhibit a rust expansion crack, the failure form of the test piece is mainly pull-out splitting failure along the rust expansion crack, as shown in Figure 6c.

### 3.3. Effect of Corrosion Degree on Ultimate Bond Strength

The average bond strength over the bond length is calculated as per Equation (3) [7]:

(3) $$\tau =\frac{F}{\pi Dl},$$

where *F* is the pull-out force; *D* is the diameter of the steel bar; and *l* is the anchorage length between the steel bar and concrete.

**Figure 8:**
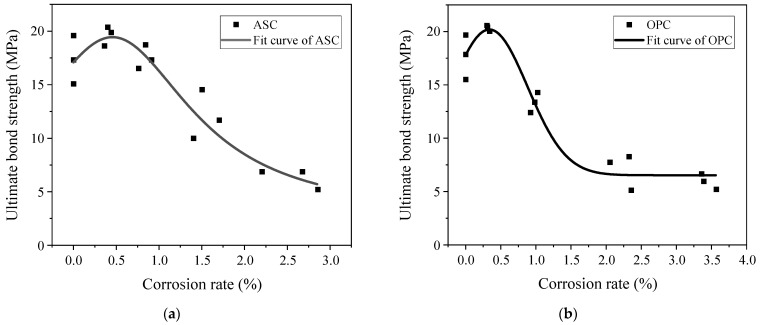
Relationship between the corrosion degree and the ultimate bond strength: (**a**) ASC and (**b**) OPC.

The test data obtained through the pull-out test were examined, and the results are shown in Table 6. The ultimate bond strength for the specimens with corrosion cracking is the corresponding bond strength when the specimens can bear the maximum pull-out force. The ultimate bond strength changes with the corrosion degree for both ASC and OPC specimens. For the specimens with corrosion cracking, the bond stress is significantly reduced. Figure 8 shows the relationship between the corrosion degree and the ultimate bond strength of the ASC and OPC test pieces.

As can be observed from Figure 8, for both ASC and OPC, the bond stress at the ultimate stage increases first and then decreases with increasing corrosion degree, and its value reaches the maximum when the designed corrosion degree is 0.5%. At lower corrosion degrees (≤0.5%), the ultimate bond stress increases with corrosion. This is due to the filling up of the bond interface with corrosion products. Furthermore, the steel bar’s corrosion increases its surface roughness and improves the friction force between the steel and the concrete. Another possible explanation for the bond improvement is that when the corrosion degree of the steel bar is low, the hydration reaction is still carried out inside the test piece in sodium chloride solution; thus, when chloride ions invade into the concrete, they could react with the hydration products, and their reacted products could enhance the pore structure and the compressive strength of the concrete. When the corrosion degree of the steel bar increases above 0.5%, corrosion products increase. This leads to a decrease in the mechanical interlock force between the concrete and the steel. As the corrosion degree further increases, the stress generated by the corrosion products leads to the cracking of the concrete, thereby reducing the ultimate bond stress. The cracks extend to the surface of the specimen with an increase in corrosion degree. After the specimen is cracked, the mechanical interlock force is significantly reduced.

Figure 8 shows that the value of the bond gradually decreases with increasing corrosion degree after reaching the maximum value. The maximum bond stress is attained at about 0.5% for both ASC and OPC. However, after 0.5% corrosion degrees, the OPC specimens’ bond drops much faster than the ASC specimens. After a quick drop, the bond becomes almost constant, starting from a corrosion degree of 2%. However, in the case of ASC, similar to other research [16], the bond decreases constantly with an increase in the corrosion degree.

Table 7 summarizes the average bond stress values at each corrosion degree to better compare ASC and OPC. In this table, n is the ratio of OPC’s ultimate bond stress to that of ASC.

Table 7 shows that when the designed corrosion degree is between 0 and 0.5%, the average ultimate bond strength of the OPC specimens is slightly higher than that of the ASC specimens. When the designed corrosion degree is between 1% and 2%, the average ultimate bond of the ASC specimens is much higher than that of the OPC specimens. Both the ASC specimens’ and the OPC specimens’ bonds have reduced at these corrosion degrees. However, the OPC specimens’ reduction is more significant. This is because when the designed corrosion degree of the OPC specimens is 1%, some specimens have cracked, indicating that the bonding interface of the OPC specimens has been damaged. This weakens the interlocking force of the concrete to the steel bar and reduces the bond strength. For the ASC specimens, when the corrosion degree is 1%, the bond strength does not decrease significantly. Compared to the non-corroded specimens, the ASC specimens still have strong bond strength. When the corrosion degree is 2%, all the OPC specimens are cracked, and the confinement provided by the concrete is significantly reduced, which affects its bond strength. However, at this corrosion degree, the ASC specimens do not crack. Although the friction force between the steel bar and the ASC has increased, the mechanical interlock force between the two has reduced. The increased value in the former is far less than the decreased value in the latter, so the bond strength is still reduced. When the designed corrosion degree is 3%, the cracks on the surface of the OPC specimens become wider, and the ultimate bond strength continues to decrease due to the increase in the corrosion degree of the steel bar. The ASC specimens are cracked, and the confinement force of the concrete decreases, resulting in a rapid decrease in the ultimate bond strength.

### 3.4. Effect of Corrosion Degree on Initial Bond Strength

**Figure 9:**
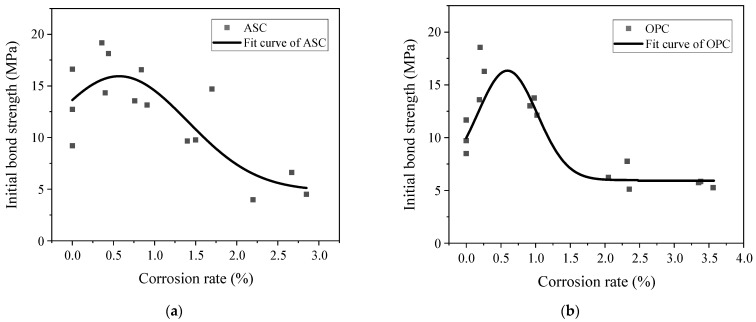
Corrosion degree and initial bond strength: (**a**) ASC and (**b**) OPC.

The initial bond strength is the bond strength at the endpoint of the elastic-plastic stage of the bond–slip curve. These initial bond strength values are summarized in Table 8. Here, the free end slip of the steel bar is defined as 10 μm [28]. Figure 9 shows the relationship between the corrosion degree and the initial bond strength of the OPC and ASC specimens.

**Figure 10:**
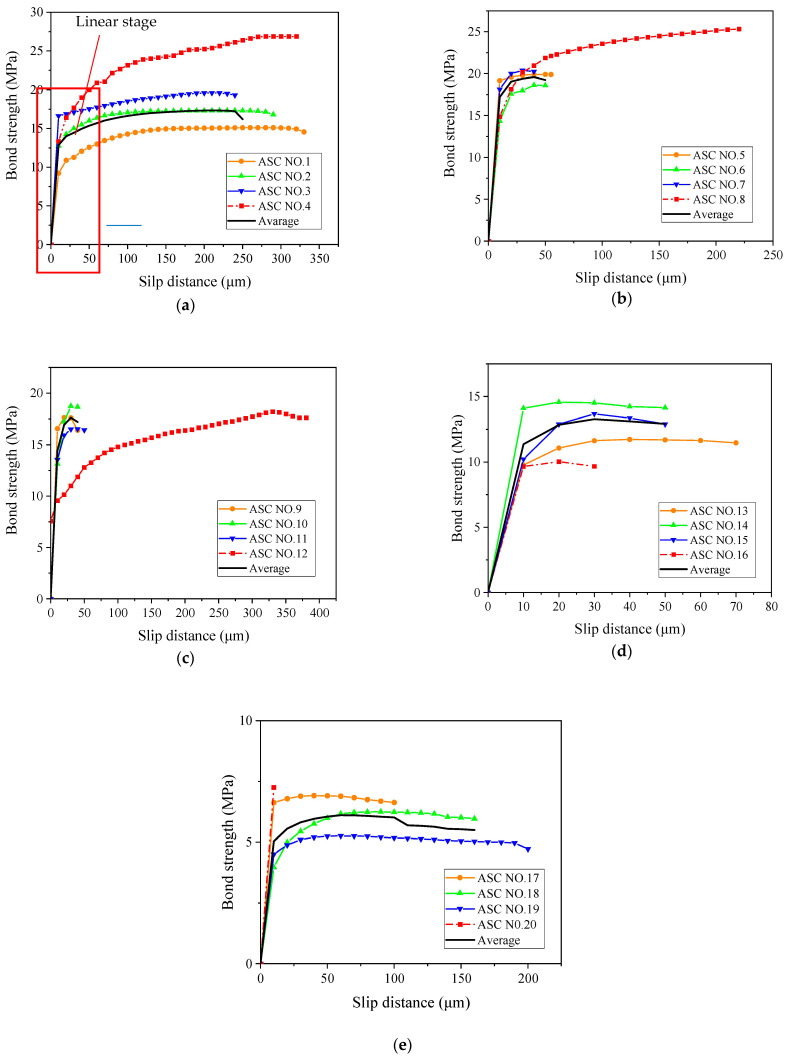
ASC bond–slip curve (actual corrosion degree): (**a**) control; (**b**) 0.4%; (**c**) 0.83%; (**d**) 1.53%; and (**e**) 2.57%.

Similar to the ultimate bond stress, the initial bond stress values first increase and then decrease, as can be observed in Figure 10. When the corrosion degree is low, although the chemical cementation force is broken, the expansion force generated by the thin corrosion layer on the surface of the steel bar does not damage the bonding interface; rather, it enhances the interaction force between the two and improves the bond. When the corrosion degree is high, the thick corrosion layer on the surface of the steel bar reduces the friction coefficient. At the same time, the expansion force generated by the corrosion products destroys the bonding interface, causing the crack to extend from the inside of the concrete to the surface of the specimen and weakening the bond. The test data in Table 8 were examined; each specimen’s average initial bond strength was calculated, and the results are shown in Table 9.

As shown in Table 9, when the designed corrosion degree is 0.5%, the initial bond stress of both ASC and OPC reaches the maximum value. In the case of a lower designed corrosion degree (≤1%), the steel bar corrosion still results in a higher bond stress value for both ASC and OPC. After the corrosion cracking of the specimens, the initial bond stress decreases sharply. It is also seen in the figure that the initial bond stress of the ASC specimen is higher than that of the OPC specimen, and the ASC specimen shows higher bond values at most corrosion degrees except at 3%.

### 3.5. Effect of Corrosion Degree on Average Bond Strength

**Figure 11:**
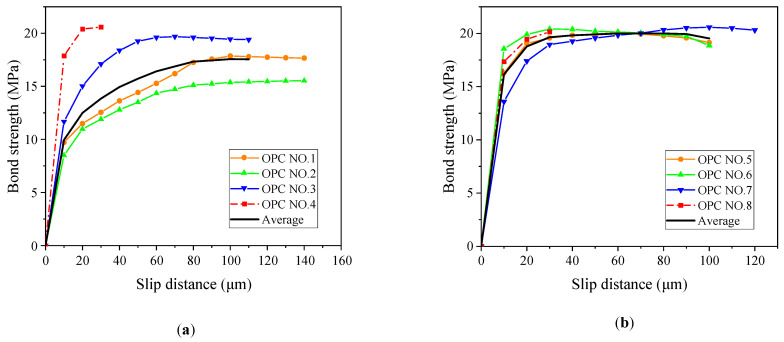

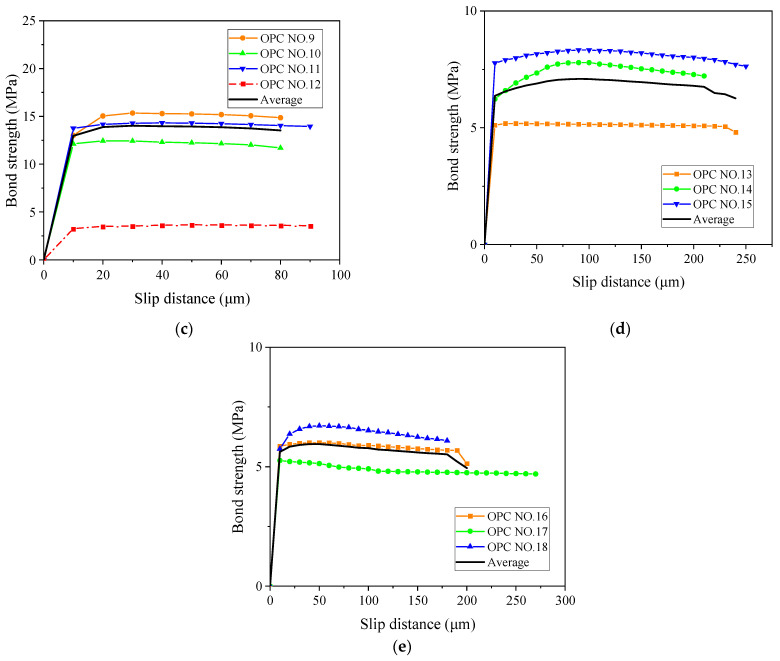
OPC bond–slip curve (actual corrosion degree): (**a**) control; (**b**) 0.22%; (**c**) 0.97%; (**d**) 2.24%; and (**e**) 3.43%.

Figure 10 and Figure 11 show the bond–slip curves for the ASC and OPC specimens, respectively. The slip is the amount of the free-end displacement of the steel bar. The data were recorded by recording the pull-out force at every 10 μm of slip, and the bond was then calculated as per Equation (3).

**Figure 12:**
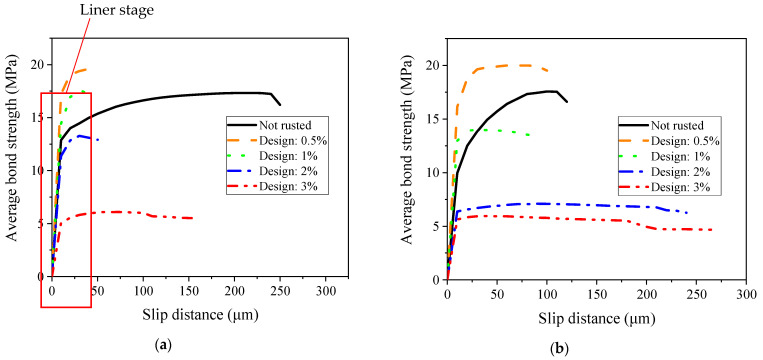
Average bond–slip relationship: (**a**) ASC and (**b**) OPC.

According to the test data in Figure 10 and Figure 11, the average bond strength of the two kinds of concrete under each corrosion degree was calculated, and the average bond–slip curve was drawn, as shown in Figure 12.

The bond–slip curve, up to the maximum bond, can be divided into two main regions for all the specimens. The first region is characterized by high stiffness and can be taken to be linear. The slope of this linear branch is called bond stiffness [21]. In the second region, the bond increases nonlinearly up to the maximum value. It can be seen from Figure 12 that although the bond strength of the ASC specimens is similar to that of the OPC specimens at each corrosion level, the bond stiffness of the ASC specimens is much higher than the OPC specimens. This finding is consistent with other research conducted on non-corroded steel bar [7,16,27]. Depending on the failure mode, the nonlinear part ends with a softening branch or a sudden drop.

As can be observed in Figure 10, Figure 11 and Figure 12, the change in the corrosion degree affects the slip. A comparison of the control and the 0.5% corrosion degree shows that the linear region significantly increases with corrosion (see Figure 10 and Figure 11). This is due to the increased bonding of the steel and the concrete due to the presence of some corrosion products. As the corrosion degree further increases, the nonlinear part of the bond–slip curve approaches an almost flat curve. At the highest corrosion degree, one can observe that the bond stress changes from linear to an almost constant bond value suddenly. This is because of the crack on the surface of the specimen. The steel bar is pulled out slowly with the crack widening, so the slip amount increases without an increase in force.

At a slip of 25 μm, the average bond strength, from large to small, for the ASC specimens is in the order of 0.5%, 1%, 0%, 2%, and 3%, while for the OPC specimens, it is in the order of 0.5%, 0%, 1%, 2%, and 3%. This shows that the ASC specimens have better corrosion tolerance than the OPC specimens. In a previous study [29] that used the chloride penetration test and mercury pressure test, the authors found that ASC has better chloride ion erosion resistance and compactness of pore structure.

### 3.6. Comparison of Bond–Slip Curves between ASC and OPC

**Figure 13:**
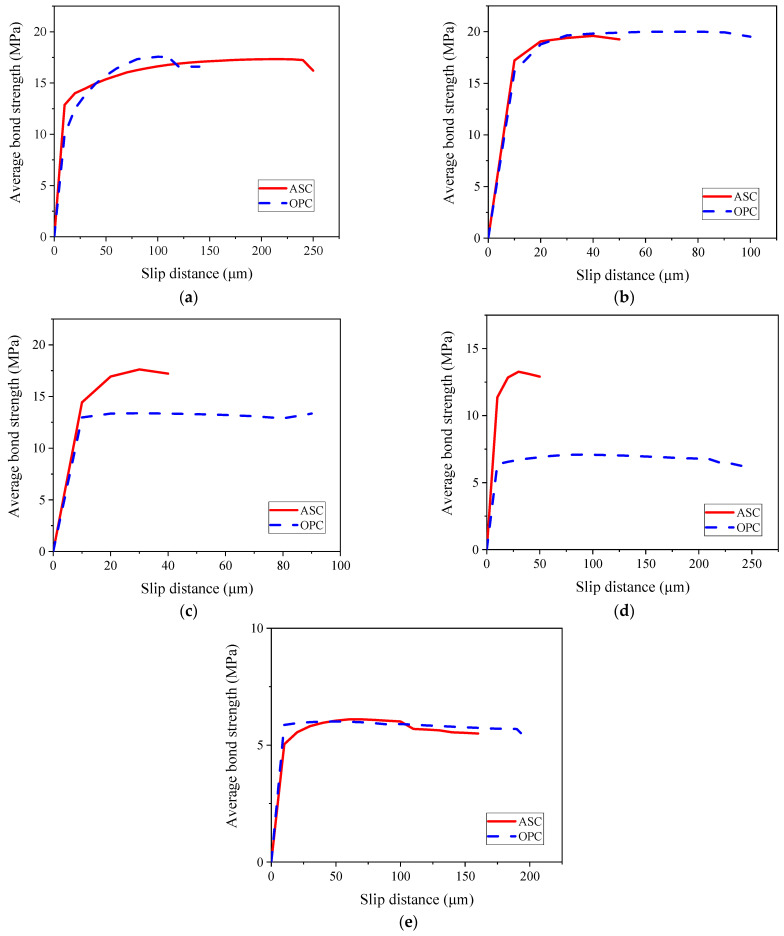
Comparison of bond–slip curves for ASC and OPC: (**a**) control; (**b**) 0.5% corrosion degree; (**c**) 1% corrosion degree; (**d**) 2% corrosion degree; and (**e**) 3% corrosion degree.

Figure 13 shows the bond–slip relations of the ASC specimens and OPC specimens. The curves shows that the bond–slip relations change under the influence of the corrosion of steel bar. Figure 13a indicates that the ASC and OPC specimens show similar bond strengths because their steel bar is not corroded, and the compressive strengths of the two types of concrete are similar. When the corrosion degree of the steel bar is low, the bond strength of the two kinds of concrete improves, and the increased range is similar, as shown in Figure 13b. When the designed corrosion degree is 1%, the bonding strength of the ASC specimen is higher than that of the OPC specimen. As shown in Figure 13c, the designed corrosion degree of the steel bar is 1% in the actual test, and the surface of the existing OPC test piece is corroded and cracked. The reason may be that an increase in the corrosion degree of the steel bar destroys the bonding interface between the two, causes cracks in the concrete, weakens the binding effect of the steel bar, and leads to a sharp decrease in the bonding strength. Compared to the non-corroded specimens, the ASC specimens are affected by corrosion and can still maintain a strong bond strength.

When the designed corrosion degree of the steel bar reaches 2%, the bonding strength of the ASC specimen is higher than that of the OPC specimen. At this corrosion degree, the OPC specimen is corroded and cracked. Hence, the bond strength drops sharply. The ASC specimen, on the other hand, is not corroded and cracked. Therefore, compared to the OPC specimen, the ASC specimen has a stronger bond. When both concretes are corroded and cracked due to the steel bar’s corrosion, they show similar bond strength again, as shown in Figure 13e.

## 4. Conclusions

The bond performance of corroded and non-corroded steel bars embedded in OPC and ASC was investigated using an accelerated corrosion test and a pull-out bond test. Different corrosion degrees were taken as the parameters. For each specimen, the failure mode of bond, the average bond strength, the slip at the free ends, and the bond–slip relationship were determined and compared. The results of this study can be summarized as follows:

(a) The steel bars were corroded earlier when embedded in OPC. When the corrosion degree of the OPC specimens was approximately 1%, corrosion cracking occurred. The ASC specimens showed corrosion cracking later when the corrosion degree was 2%; this is because of the superior chloride resistance and the compactness of the pore structure of ASC.
(b) The ultimate and initial bond stresses increased with the corrosion degree, up to 0.5%, and then decreased afterward.
(c) The bond strength between ASC and OPC and steel bar is similar for the non-corroded steel bars and at low corrosion levels. As the corrosion degree increased above 0.5%, the OPC specimens showed a sudden drop in bond, while the ASC specimens showed a gradual decrease.
(d) At a 1% corrosion degree, the OPC specimen showed a lower bond strength than the control, while the ASC specimen showed a slightly higher value than the control. This shows the better tolerance of ASC for corrosion.

## Figures and Tables

**Table 1:** The chemical composition of slag and cement (mass %).

Composition	CaO	SiO_2_	Al_2_O_3_	MgO	SO_3_	Fe_2_O_3_	K_2_O	Others
GGBS	38.95	33.35	17.96	6.02	1.39	0.382	0.318	1.63
Cement	57.1	21.6	7.12	4.15	3.06	4.43	0.721	1.779

**Table 2:** Physical and chemical indices of water glass.

Water Glass	Na_2_O (%)	SiO_2_ (%)	Baume Degrees	Modulus	Transparency
	26.96	9.02	39.5–40.2	3.07	≥82

**Table 3:** Technical index of NaOH (analytical purity).

NaOH	Purity	Carbonate	Chloride	Sulfate	Al	Ca
Content (%)	≥99.5	≤1.5	≤0.005	≤0.005	≤0.002	≤0.01

**Table 4:** Concrete mix proportion (kg/m^3^).

Material	Cement	Water	Coarse Aggregate	Sand	GGBS	Activator	Compressive Strength
OPC	450	157.5	1125	675	-	-	61.0 ± 2.4
ASC	-	-	1074	716	400	184	64.1 ± 1.3

**Table 5:** Designed and measured values of steel bar corrosion degree in OPC.

ASC	Design Corrosion Degree (%)	Measured Corrosion Degree (%)	Average Corrosion Degree (%)	OPC	Design Corrosion Degree (%)	Measured Corrosion Degree (%)	Average Corrosion Degree (%)
1	0	0	0	1	0	0	0
2	0	0		2	0	0	
3	0	0		3	0	0	
4	0.5	0.4	0.4	4	0.5	0.26	0.22
5	0.5	0.44		5	0.5	0.2	
6	0.5	0.36		6	0.5	0.19	
7	1	0.84	0.84	7	1	0.92	0.97
8	1	0.91		8	1	1.02	
9	1	0.76		9	1	0.98	
10	2	1.5	1.53	10	2	2.05 *	2.24
11	2	1.7		11	2	2.32 *	
12	2	1.4		12	2	2.35 *	
13	3	2.2 *	2.57	13	3	3.56 *	3.43
14	3	2.85 *		14	3	3.35 *	
15	3	2.67 *		15	3	3.38 *	

* Cracked due to corrosion.

**Table 6:** Corrosion degree and ultimate bond strength of OPC.

ASC	Corrosion Degree (%)	Ultimate Bond Strength (Mpa)	OPC	Corrosion Degree (%)	Ultimate Bond Strength (Mpa)
1	0	17.11	1	0	15.52
2	0	18.60	2	0	17.85
3	0	15.11	3	0	19.68
4	0.4	18.63	4	0.26	20.04
5	0.44	20.24	5	0.2	20.03
6	0.36	19.88	6	0.19	20.57
7	0.84	17.62	7	0.92	13.40
8	0.91	18.74	8	1.02	12.44
9	0.76	16.54	9	0.98	14.31
10	1.5	11.733	10	2.05 *	7.79
11	1.7	14.56	11	2.32 *	8.32
12	1.4	10.02	12	2.35 *	5.18
13	2.2 *	6.24	13	3.56 *	5.26
14	2.85 *	5.25	14	3.35 *	6.71
15	2.67 *	6.91	15	3.38 *	6.01

* Cracked due to corrosion.

**Table 7:** Designed corrosion degree and average ultimate bond strength.

Designed Corrosion Rate (%)		0	0.5	1	2	3
	Ultimate Bond Strength (MPa)					
ASC		17.34	19.62	17.54	12.1	6.36
OPC		17.68	20.34	13.38	7.09	5.99
n		1.01	1.03	0.76	0.58	0.94

n is the ratio of OPC’s ultimate bond stress to that of ASC.

**Table 8:** Corrosion degree and initial bond strength.

ASC	Corrosion Degree (%)	Initial Bond Strength (MPa)	OPC	Corrosion Degree (%)	Initial Bond Strength (MPa)
1	0	12.73	1	0	9.71
2	0	16.62	2	0	8.49
3	0	9.21	3	0	11.67
4	0.4	14.33	4	0.26	16.28
5	0.44	18.13	5	0.2	18.55
6	0.36	19.17	6	0.19	13.59
7	0.84	16.57	7	0.92	13.01
8	0.91	13.15	8	1.02	12.12
9	0.76	13.55	9	0.98	13.75
10	1.5	9.77	10	2.05 *	6.22
11	1.7	14.69	11	2.32 *	7.76
12	1.4	9.66	12	2.35 *	5.11
13	2.2 *	3.97	13	3.56 *	5.26
14	2.85 *	4.5	14	3.35 *	5.74
15	2.67 *	6.62	15	3.38 *	5.84

* Cracked due to corrosion.

**Table 9:** Designed corrosion degree and average initial bond strength.

Design Corrosion Rate (%)		0	0.5	1	2	3
	Ultimate Bond Strength (MPa)					
ASC		12.85	17.21	14.42	11.17	5.03
OPC		9.95	16.14	12.96	6.36	5.62
m		0.77	0.93	0.89	0.56	1.11

m is the ratio of OPC’s initial bond stress to that of ASC.

## Data Availability

For the access to the data involved in this research, please contact the corresponding author Yifei Cui via: cuiyifei@qut.edu.cn.
